# Case of Fracture of the Anatomical Neck of the Humerus, and Recovery

**Published:** 1871-04

**Authors:** Edward Duffield

**Affiliations:** Hannibal, Mo.


ARTICLE V.—*Case of Fracture of the Anatomical Neck of the Humerus, and Recovery.* By EDWARD DUFFIELD, M.D., Hannibal, Mo.

On the morning of the third of September last (1870), the writer was summoned at 7 o'clock P. M., at the request of his friend Dr. Brittingham, to assist him, in the case of the Hon. Jno. B. H—m, an old gentleman, over 72 years of age, who had fallen and injured his shoulder, to an extent which the Dr., from the hasty examination made, was not prepared to determine. The case presents several points of such decided interest, that we shall feel warranted in giving the details at length.

It would seem, that the Judge, being in ill health, and a very rest less sleeper, was in the habit of rising at night, to walk the floor and smoke. On this occasion he had risen about 2 o'clock A. M., and placing a chair,had mounted upon it, in order to light his hall lamp; the chair lurching, pitched him upon his left shoulder, and also striking his head with sufficient force to stun him. The family, alarmed, put him back upon his bed; and not discovering any blood, or evidence of serious injury, other than the shock, did not send for their medical adviser until his moaning about six o'clock A. M., made them uneasy lest he might be more hurt than they at first supposed. The Doctor came, and from the first impression rather supposed the bone dislocated at the shoulder, and urged my being sent for. The writer found him on his back, in a semi-unconscious state, answering, yet indistinctly, when spoken sharply to, with his right hand firmly grasping his left wrist, the elbow in almost natural position close to the body, the arm not altered in its length or proportions, with no diversion of its axis, the outline of the shoulder perfect, with neither swelling nor depression of the deltoid, or of the axillary walls, and the arm susceptible of the diverse motions, though painful when moved, and with the head of the humerus distinctly felt in the glenoid cavity; and the idea of dislocation was abandoned. Having fixed the head of the humerus with my left hand, and seizing his left wrist, and pushing it firmly against the socket, and rotating at the same moment, a distinct, clear crepitus was heard and could be repeated; holding it thus fixed at head and rotated, by separating the muscles with the fingers, a lack of coaptation was discoverable, but not so when the arm was released and placed as at first; and the diagnosis was made of fracture of the humerus at the anatomical neck, and at once coincided in by Dr. Brittingham. The age, and the peculiar previous condition, combined to render this a grave and but slightly hopeful case; indeed, with but faint prospect of having any future use of the arm, admitting that life was spared.

The patient had been in ill health for two or three years, though previously a man of stalwart frame and vigorous constitution. About ten months immediately preceding this hurt, he had been seized with an incomplete hemiplegia of his right side, not entirely destroying voluntary motion, but seriously impairing his memory, and suspending wholly his power of articulation. Even after the entire recovery of the voluntary power of motion in his limbs, the memory and speech remained almost as bad as at first. We say almost, for there were times when the memory seemed to light up, and his power of expression would improve for a few days, but only to relapse. At the moment of the injury, whilst his memory was improving, yet his speech was at times almost indistinguishable—points to be noted for reasons given later. During these few months he was very anæmic; irritable, appetite poor, bowels torpid, tongue coated densely with a closely adherent dirty brown coating, secretions scant, and himself very feeble, with considerable bronchial irritation and some cough, as Drs. B. and D., his former advisers, informed me, the writer not seeing him. This peculiar combination, as remarked, did not serve to encourage us, but something had to be done. The arm had not yet swelled, and neither did it, or but triflingly, during the progress of the case. We cut and fitted a stout sole leather splint to the shoulder and along the whole length of the arm, moulding it easily, after soaking in warm water, padding with wool carded, (which we prefer, on account of more elasticity, to the cotton), over the projections of the elbow and wrist; and over this was applied firmly, yet carefully, a bandage, with the latter and the splint, so arranged at points as to apply arnica, camphor, etc., when needed. The arm was slightly flexed at the elbow and placed upon a pillow, and padded around lightly to support it; and careful watching enjoined upon his attendants to see that it was not moved, except when we were present to direct it.

For six days he lay in a semi-stupor; partially conscious when aroused and his attention fixed, but sleeping stertorously when left to himself. During this time he was given freely beef tea, rich milk, brandy, etc., and the carbonate of ammonia, under which, for the first time since his paralysis, his tongue cleaned, and under the use of it and bitter infusions, he became conscious, *memory aroused*, *speech clear*, appetite improved—and with these an inordinate craving for spirits and cigars. We forbade the latter, entirely, giving him brandy, only as it seemed required by circumstances. In our absence he would manage, through servants or others, to get his cigars, and after smoking he was invariably betrayed, by the presence of hiccough, often prolonged to exhaustion. When he suffered from pain or sleeplessness, the hydrate of chloral in ten grain doses, seemed to exert a happy influence over him. As he rallied, we gave him iron and quinine, and a more liberal diet of beefsteak, game, mutton chops, soft eggs, fruit, etc. The family and friends remarked from the moment of his fully returned consciousness, the restored memory and improveds peech; he recalled incidents to them forgotten before his paralysis, and inclined to be very talkative and pleasant, as formerly.

It was but a short time from his resumption of speech, until the writer discovered that he was aphasic and called the attention of Dr. B. to it, in order to the ascertaining whether this was the case antecedent to the fracture or not, but the Doctor had not noticed, nor had his family—in fact, they had never thought of it until attention was called to it; and it was a matter of regret to me to be unable to decide whether his aphasia was the result of his fall, or previous paralysis. Certainly the fall seemed to awaken his dormant memory and to restore his suspended speech; we say suspended, so imperfect was it before. In arranging his business affairs, he recalled incidents, anterior to his paralysis, which even his lawyers had forgotten, and yet were essential to the management of his large estate; and indeed so accurate in minutest details was this restored memory, that when his agents would doubt or contradict, he would prove himself right by reference to the documentary evidence or living witnesses. Yet when speaking, his substitution of words for each other, or his forgetfulness of them entirely, would be noticed by himself, when he would laughingly say: "I know what I want to say, but can't hit the word"! The idea would be there, but often laughably mixed in expression. He would always speak of "his leg being broken at the shoulder," never calling it arm; and if reminded of it, in a moment would say leg for arm again. He would say "that his broken leg was sore, so that he could'nt move his hand"! He would ask his wife for "a —— to put the fire of his smoke in," meaning a waiter for the ashes of his cigar. And when any one was writing for him, and he would halt at a word, he would say, "you know what I mean," and on being told, would go on until brought to a halt again. He observed it himself and would often laugh at it, saying, "my mind is all right, but the words quit me"!

The splint was kept on for seven weeks, and when removed we found, in spite of our care, two ulcers—one on the elbow, the other on the wrist; these, however, while somewhat tedious, healed kindly; and certainly no persons could be more surprised and gratified to find the fracture firmly knit, without deformity or lameness. Whether or not the union is bony, let others decide; we know that it is now perfectly solid and unyielding, and that the patient, though otherwise very feeble and infirm, has in all particulars excellent use of his arm; feeding himself, and lifting readily and securely with it—in fact, moves it in all directions, and it is unyielding at the former point of fracture.

We inclined to the opinion, then and now, that the line of fracture extended outside the capsular ligament; and how much influence, if any, was exerted by this in the healing, we are not prepared to affirm. When we recapitulate the circumstances, of the fracture of the anatomical neck in a paralytic of 72 years of age, anæmic and enfeebled, and consider the chances of securing any union, it seems as though hopeless, and certainly encourages us not to despair under the most adverse circumstances.

It may be asserted that we may have been mistaken in the fracture, or, at least, in its character. As to the first, we know we were not—it was too palpable; and as to the second, it may be possible, yet we think not, and we are supported in this by the peculiar diagnostic symptoms given. Had there been a lack of coaptation at rest, or a difficulty of preserving it at any time; or had the elbow hung away from the body when he was raised, and the arm left pendant; or had the shaft and head of the bone moved separately, and not synchronously, why we might possibly have divined a different fracture—that of the surgical neck, perhaps—but none of these things were found, or not enough, at least, united to alter our opinion.

It was the second case of fracture of the anatomical neck of the humerus we had ever seen, and presented several salient points of such decided interest to my own mind, that it seemed entirely possible it might interest others no less; and hence the space taken up in reporting it.

